# High Levels of Expression of Cartilage Oligomeric Matrix Protein in Lymph Node Metastases in Breast Cancer Are Associated with Reduced Survival

**DOI:** 10.3390/cancers13235876

**Published:** 2021-11-23

**Authors:** Konstantinos S. Papadakos, Catharina Hagerling, Lisa Rydén, Anna-Maria Larsson, Anna M. Blom

**Affiliations:** 1 Department of Translational Medicine, Division of Medical Protein Chemistry, Lund University, 214 28 Malmö, Sweden; konstantinos.papadakos@med.lu.se; 2 Department of Laboratory Medicine, Division of Clinical Genetics, Lund University, 221 85 Lund, Sweden; catharina.hagerling@med.lu.se; 3 Department of Clinical Sciences, Lund Division of Oncology, Lund University, 221 84 Lund, Sweden; lisa.ryden@med.lu.se (L.R.); anna-maria.larsson@med.lu.se (A.-M.L.)

**Keywords:** COMP, metastatic patients, serum-COMP, lymph node metastases

## Abstract

**Simple Summary:**

Cartilage oligomeric matrix protein (COMP) is an emerging independent prognostic marker for breast cancer patients. COMP expression by cancer cells affects their metabolism, metastases, and the abundance of cancer stem cell populations. This study assessed the levels of COMP in the sera of metastatic breast cancer patients. Further, matched tumor tissues from the primary tumor and metastases were stained for COMP expression with immunohistochemistry. The levels of serum COMP were highest in the blood of metastatic ER-positive and HER2-positive patients. The expression of COMP in primary tumors correlated with COMP expression in the metastatic loci. Lymph node metastases (LNM) with COMP expression were associated with reduced survival. The expression of COMP in LNM at the time of primary diagnosis could indicate later development of visceral and lung metastases.

**Abstract:**

Cartilage oligomeric matrix protein (COMP) is a regulator of the extracellular matrix and is expressed primarily in the cartilage. Recently, COMP expression was also documented in breast cancer patients both in sera and tumor biopsies, in both of which it could serve as an independent prognostic marker. This study aimed to assess COMP as a potential biomarker in the group of metastatic breast cancer patients. Levels of COMP were measured by ELISA in serum samples of 141 metastatic breast cancer patients. Biopsies from primary tumors, synchronous lymph node metastases, and distant metastases were stained for COMP expression. The levels of serum COMP were higher in patients with ER- and HER2-positive tumors when compared to triple-negative tumors and correlated with the presence of bone and lung metastases, circulating tumor cell count, and clusters. Most of the primary tumors expressing COMP (70%) retained the expression also in the lymph node metastases, which correlated with visceral metastases and reduced survival. In conclusion, COMP appears as a valuable biomarker in metastatic breast cancer patients indicating a more severe stage of the disease. Serum COMP levels were associated with specific types of metastases in patients with metastatic breast cancer emphasizing that further studies are warranted to elucidate its potential role as a monitoring marker.

## 1. Introduction

Cartilage oligomeric matrix protein (COMP) has been traditionally viewed as an extracellular matrix (ECM) protein that is expressed by chondrocytes of cartilage [1] as well as fibroblasts under fibrotic conditions [2] and contributes to the organization of ECM [3]. COMP is also known as thrombospondin 5, belonging to the thrombospondin family [4]. Recently, several new functions have been ascribed to this large, pentameric molecule. Thus, COMP was shown to contribute to vascular homeostasis since its degradation by ADAMTS-7 [5] regulates vascular remodeling. COMP has also been found in atherosclerotic plaques [6] and lesions contributing to restenosis of the artery [7]. Furthermore, COMP inhibits thrombin [8] and can act as a regulator of the complement system [9,10].

Moreover, COMP has been found in several cancer types in which it is expressed by epithelial cancer cells and in the surrounding matrix. This has so far been observed in breast- [11], prostate- [12], colon- [13,14], and hepatocellular-carcinoma [15]. Most importantly, in all of the types of cancer that have been studied so far, high expression of COMP has been correlated with reduced survival of the patients. Our previous studies in primary breast cancer revealed that COMP expression in breast tumor cells was correlated with reduced breast cancer-specific survival and recurrence-free survival as an independent prognostic marker [11]. Further, COMP expression in cancer cells correlated positively with the presence of lymph node metastases and estrogen/progesterone receptor positivity. We also found increased levels of COMP in sera of metastatic breast cancer (MBC) patients compared with those with early breast cancer. Further, there was correlation between the serum levels of COMP and the presence of liver and bone metastases. Elevated serum levels of COMP appeared to serve as an independent prognostic marker of survival for the metastatic patients [16].

The mechanisms underlying the effects of COMP in breast cancer progression are under investigation. It has been already established that COMP expression in cancer cells increases their invasive potential, alters metabolism (Warburg effect), and renders them resistant to apoptosis [15]. Further, COMP facilitates the interaction between Notch3 and its ligand Jag1, which leads to the generation of a larger population of cancer stem cells [17].

The goal of the current study was to further evaluate COMP as a potential biomarker in metastatic breast cancer patients by assessing the serum COMP levels and COMP expression in relation to the severity of the disease and outcome.

## 2. Materials and Methods

### 2.1. Cohort Description

Patients with newly diagnosed MBC were included in a prospective monitoring trial (ClinicalTrials.gov NCT01322893) before the start of systemic therapy. The inclusion criteria were MBC diagnosis, age > 18 years, Eastern Cooperative Oncology Group (ECOG) performance status 0–2, and predicted life expectancy > 2 months. Serum samples were taken at a baseline, before the start of systemic therapy and stored at a biobank. Circulating tumor cells (CTCs) were enumerated using the CellSearch system. Clinicopathological data and monitoring of patients were prospectively collected in case report forms. Included patients received systemic therapy according to national clinical guidelines. A response evaluation was performed approximately every third month and the progression versus non-progression was defined according to a modified RECIST criteria [18]. Initial results from the study have previously been published [19].

### 2.2. Determination of COMP Levels in Sera (S-COMP)

The S-COMP serum levels were measured using an IVD approved ELISA (AnaMar AB, Immunodiagnostic systems) following manufacturer instructions. In brief, serum was diluted (1/10) in the provided sample buffer, added to a precoated 96-well plate together with an enzyme conjugated antibody, and incubated for 2 h at room temperature. The plates were then washed 6 times, developed with TMB substrate, and measured at 450 nm using Cytation 5 (Biotek, Winooski, VT, USA).

### 2.3. Immunohistochemical (IHC) Detection of COMP in Tumor Tissues

Breast cancer tissues were mounted in a tissue microarray. Antigen retrieval was performed with the Envision Flex high pH kit (Agilent-Dako, Santa Clara, CA, USA) using a PT-link module (Agilent-Dako). The tissues were stained with 0.47 μg/mL rabbit polyclonal affinity purified anti-COMP in-house antibody that was previously evaluated for its specificity [11], utilizing Envision Flex (Agilent-Dako) reagents in the Autostainer Plus system according to the manufacturer’s protocol (Agilent-Dako). Slides were scanned with Aperio Scanner system (Leica) at 40× and the intensity of COMP was evaluated independently by two experienced researchers in a blinded fashion using scores: 0 for negative staining, 1 for low expression, 2 for moderate expression, and 3 for high expression. Staining in the tumor cells was evaluated separately from stroma.

### 2.4. Statistical Analyses

For comparison of clinicopathological variables, a Pearson’s chi-squared test or a Pearson’s chi-squared test for trend was used. The comparison of COMP expression status between primary tumors, LNM and DM, was performed using the McNemar’s test. Kaplan–Meier curves and log rank tests were used to illustrate and compare survival. Time to progression or death of any cause was calculated from the baseline and patients without events were censored at the last follow-up. The median follow-up time was 49 (27–93) months. All of the statistical analyses were performed using SPSS statistics (version 26.0, IBM Corp., Armonk, NY, USA).

## 3. Results

### 3.1. Serum COMP Levels in Patients with Metastatic Breast Cancer and Associations to Clinicopathological Variables

**Figure 1:**
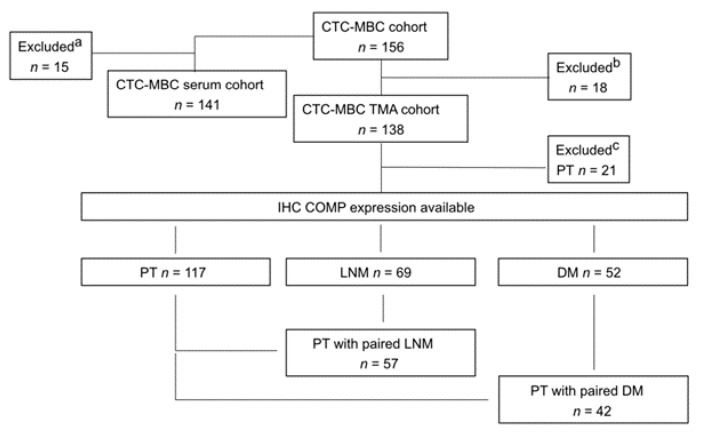
Flowchart of the study cohort. ^a^ Patient excluded due to lack of available serum samples. ^b^ Patients excluded due to lack of available tumor tissue. ^c^ Patients excluded due to lack of available primary tumor tissue for staining.

Serum samples that were taken before the start of the systemic therapy were available for 141 patients, as illustrated in the flowchart (Figure 1). Of these 141 patients, 98 had ER-positive (HER2-negative) disease, 15 had HER2-positive (ER-positive or negative) disease, and 25 were diagnosed with triple-negative breast cancer (TNBC). A total of 86 patients had a metastasis-free interval (MFI) of >3 years, 26 had an MFI ≤ 3 years, and 29 patients had de novo MBC (distant metastasis at initial diagnosis). Regarding number of metastatic loci, 98 patients had ≥3, whereas 42 patients had <3. Furthermore, 83 patients were diagnosed with visceral metastasis whereas 58 were not. A total of 72 patients had ≥5 circulating tumor cells (CTC) at baseline, whereas 13 patients had CTC-clusters. The median S-COMP level was 11.67 U/L (range: 0.00–95.24 U/L). For further analyses and correlations with clinicopathological variables, the patients were grouped by dichotomization into groups with high and low S-COMP expression, respectively, using the median as a cut-off. As illustrated in Table 1, S-COMP levels were significantly associated with age, metastasis-free interval (MFI), and histological subtype of the primary tumor. Also, high S-COMP levels were associated with HER2-positive and ER-positive disease whereas low S-COMP levels were seen in patients with TNBC. Regarding distant metastases, high S-COMP levels were significantly associated with the presence of bone metastases whereas low levels of S-COMP were seen in patients with lung metastases. Furthermore, there was a strong correlation of high S-COMP levels and CTC count (≥5CTCs), and with the presence of CTC-clusters at the baseline, where 12 of the 13 patients with CTC-clusters had high S-COMP levels.

### 3.2. Progression-Free and Overall Survival in Correlation to S-COMP Levels

**Figure 2:**
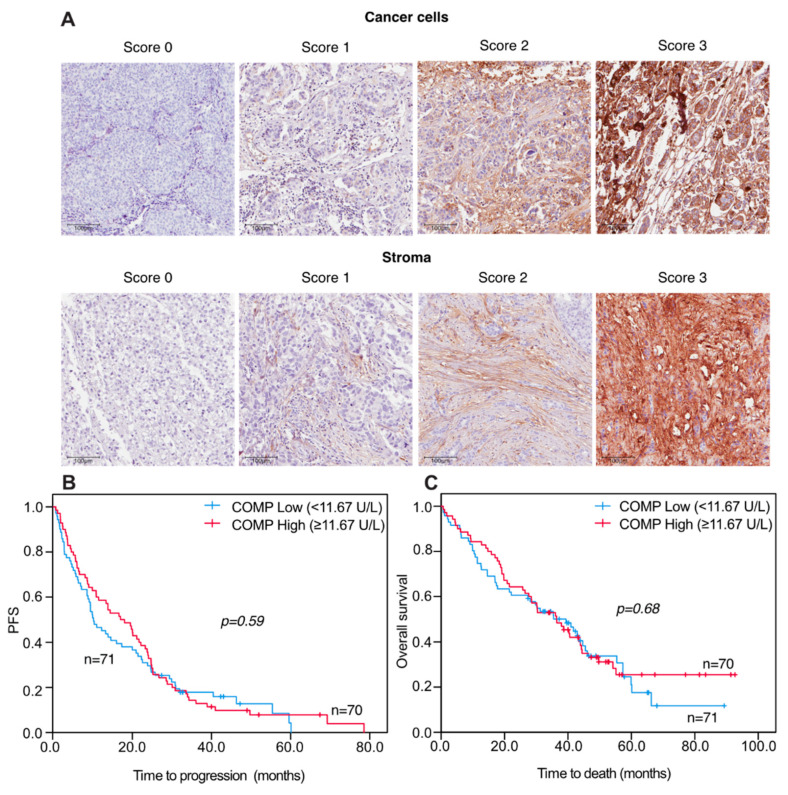
Representative pictures of TMA IHC stained for COMP expression (**A**). Progression-free and overall survival in relation to S-COMP levels in metastatic breast cancer patients Kaplan-Meier curves displaying PFS (**B**) and OS (**C**) in MBC patients with high or low S-COMP levels, using the median S-COMP level as cut-off. Scale bars: 100 μm.

When analyzing progression-free (PFS) and overall survival (OS) in relation to S-COMP levels there were no significant differences in patients with high versus low S-COMP levels, illustrated by Kaplan–Meier curves (Figure 2).

### 3.3. Correlations of S-COMP Levels and Comp IHC Expression in Distant Metastases

Comparisons of the S-COMP levels and COMP levels that were detected using immunohistochemistry (IHC expression) in cancer cells and stroma in distant metastases (DM) revealed a positive correlation of high S-COMP levels in patients that had a strong IHC COMP expression in DM stroma (*p* = 0.01; Table 1). Similarly, a trend for a positive correlation was seen between patients with high S-COMP levels and IHC COMP expression in DM cancer cells, however this was not significant (*p* = 0.13; Table 1). Comparisons of serum COMP levels to primary tumors and lymph node metastases (LNM) COMP IHC expression were not performed since the serum samples were obtained at diagnosis of metastatic disease and not at the initial breast cancer diagnosis.

### 3.4. Concordance and Discordance of IHC COMP Expression in Matched Samples of Primary Tumors, Lymph Node Metastases, and Distant Metastases

To evaluate shifts in COMP expression during tumor progression, matched pairs of primary tumors, synchronous LNM and DM, were analyzed by IHC. McNemar’s analyses were used for the evaluation of concordance and discordance of IHC COMP expression in cancer cells and stroma. As illustrated in Table 2, a significant shift was seen in the COMP IHC stroma expression from the primary tumor to LNM (*p* = 0.049), revealing that in 70% of the pairs the expression was stable but there were more pairs with a loss of COMP expression from the primary tumor to LNM than with a gain. Similarly, a trend was seen in shifts regarding COMP IHC cancer cell expression from the primary tumor to LNM, however this was not significant (*p* = 0.077). Regarding shifts from the primary tumor to DM and from LNM to DM, there were no significant shifts.

### 3.5. COMP IHC Expression and Correlations to Clinicopathological Variables

Comparisons of IHC expression in cancer cells and stroma in primary tumors and LNMs with different clinicopathological variables showed a significant correlation of COMP expression in primary tumor cancer cells with age (*p* = 0.005) and in primary tumor stroma with MFI (*p* = 0.007) (Table 3). Furthermore, there were significant correlations between high IHC COMP expression in the primary tumor cancer cells (*p* = 0.013) as well as in the primary tumor stroma (*p* = 0.013) and the presence of liver metastases. When analyzing possible correlations between LNM IHC COMP expression and clinicopathological variables there were significant correlations to performance status (*p* = 0.023) and furthermore a high COMP IHC expression in stroma correlated to visceral metastases (*p* = 0.03) and presence of lung metastases (*p* = 0.039). Associations of cancer cell and stroma COMP expression in DM showed no significant associations to the respective clinicopathological variables (Table S1).

### 3.6. COMP IHC Expression in Relation to Survival

**Figure 3:**
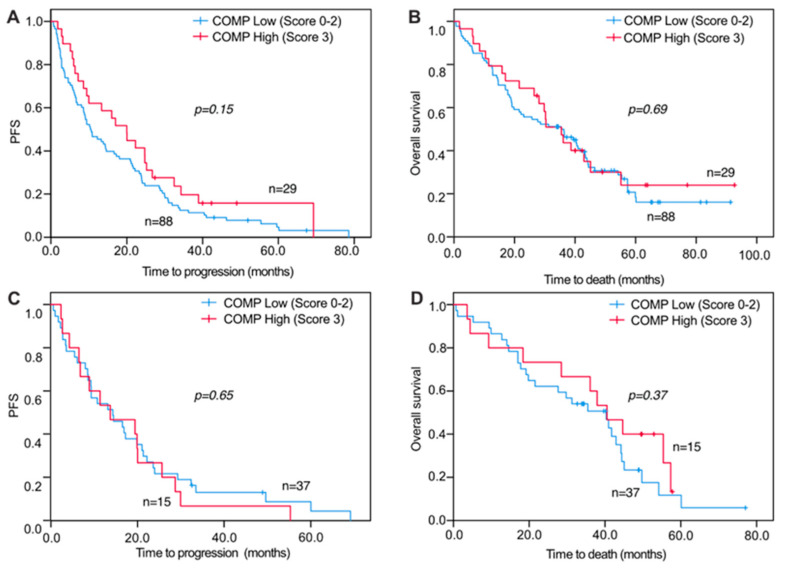
Progression-free and overall survival of metastatic breast cancer patients in relation to COMP expression in primary tumors and distant metastases. Kaplan-Meier curves displaying PFS and OS in patients with high (IHC score 3) or low (IHC score 0, 1, or 2) COMP expression in primary tumors (**A**,**B**) and distant metastases (**C**,**D**).

**Figure 4:**
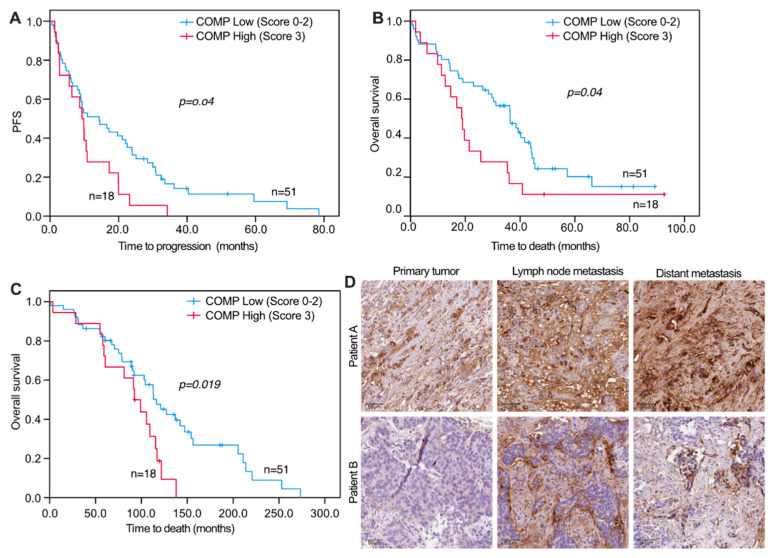
Progression-free and overall survival of metastatic breast cancer patients in relation to COMP expression in lymph node metastases. Kaplan-Meier curves displaying PFS (**A**) and OS (**B**) from the date of metastatic disease in patients with high (IHC score 3) or low (IHC score 0, 1, or 2) COMP expression in LNM. Kaplan-Meier curves displaying OS (**C**) from the date of initial breast cancer diagnosis in patients with high (IHC score 3) or low (IHC score 0, 1, or 2) COMP expression in LNM. (**D**) Representative images of IHC COMP expression from the primary tumor, LNM, and distant metastasis. The tumor of patient A retained expression of COMP in metastases while the tumor of patient B gained COMP expression in metastases. Scale bars: 50 μm.

For survival analyses, a combined variable was used that assessed the COMP IHC expression in both the cancer cells and stroma. When analyzing PFS and OS in relation to COMP IHC expression, no significant correlations were seen for primary tumors and DM COMP expression in cancer cells and stroma (Figure 3A,B). However, when analyzing COMP IHC expression in LNM cancer cells and stroma, there were significant associations with PFS and OS where a high IHC COMP expression was associated with a worse outcome (PFS, *p* = 0.040 and OS, *p* = 0.041; Figure 4A,B). Furthermore, when analyzing the survival from the date of primary tumor diagnosis, there was also a strong correlation between high COMP IHC expression in LNM and worse survival (*p* = 0.019; Figure 4C,D).

## 4. Discussion

We recently revealed that COMP levels in the serum of breast cancer patients could serve as an independent prognostic marker. Patients with metastatic disease had higher serum levels of COMP compared with those in the early stages [16]. In the current study, we investigated the associations between serum COMP levels and the clinicopathological characteristics of MBC patients and prognosis. Moreover, we assessed COMP expression by immunohistochemistry in matched pairs of primary tumors, LNM and DM, revealing that high COMP expression in LNM was associated with an inferior prognosis.

The serum level of COMP is a well-established marker of cartilage turnover [20]. Studies in rheumatoid arthritis patients revealed that patients with serum levels of COMP <12 U/L are at a lower risk of joint destruction [21,22]. In accordance with this, the median serum COMP levels in MBC patients were 11.67 U/L. This indicates that the cut-off that was used for cartilage turnover evaluation could also be applied to evaluate breast cancer patient levels of expression.

In previous studies, COMP expression by the tumor cells but not in stroma was associated with a worse prognosis of patients with early breast cancer [11,16]. COMP affects the migration, invasion, and metabolism of the breast cancer cells as well as the abundance of cancer stem cells [17]. Moreover, several studies reported similar results for prostate [12], colon [13], and hepatocellular [15] cancer patients. In the current study, high serum levels of COMP were mostly found in ER-positive and HER2-positive MBC patients. These subgroups of advanced breast cancer patients (ER^+^ or HER2^+^) are treated nowadays with targeted systemic therapy as well as chemotherapy and have a better prognosis compared to patients with triple-negative disease [23]. Thus, less aggressive strategies could be selected during therapy if a robust monitoring marker was identified. The present study is the third [11,16] showing a strong correlation between high serum levels of COMP and ER-positivity as well as an increased number of bone and liver metastases, raising concerns about managing those patients. The evaluation of serum COMP levels could potentially identify those MBC patients who have specific types of metastases and hypothetically lead to a more aggressive therapeutic strategy. Thus, longitudinal evaluation of serum COMP levels during therapy would be of interest to monitor the treatment response in patients with ER positive advanced disease to improve the tailoring of systemic therapy.

The levels of COMP in serum correlated with the deposition of COMP in the stroma of DM. A similar trend was observed between the levels of serum COMP and the expression of COMP by the cancer cells of DM. This demonstrates a correlation between the blood levels of COMP and the local expression of COMP in the distant metastasis. Furthermore, high S-COMP levels correlated with the presence of bone metastases, indicating a possible role of COMP in bone metastatic progression of breast cancer.

A significant association between serum COMP and the number and clusters of CTCs was found, supporting the hypothesis that serum COMP can be a marker of a more severe stage of metastatic disease. The presence of ≥5 CTCs carries a strong prognostic value in MBC and the presence of CTC-clusters indicates an even worse prognosis [19]. CTC-clusters have been shown to have a much higher metastatic potential compared to single CTCs [24] and the strong correlation of high S-COMP levels and CTC-clusters suggests COMP as being involved in metastatic progression. If CTCs and CTC-clusters express COMP or can secrete it into the circulation is currently not known but would be interesting to explore in future studies.

One advantage of the current study is that it includes matched tumor tissue that was collected from the same patient from both the primary tumor, synchronous LNM and DM. Analysis of this material revealed that most of the LNM cells retained the expression of COMP as identified in the primary tumor in both the cancer (73%) and stromal cells (70%). From the remaining paired samples, more LNM lost the expression of COMP (21% and 23%, respectively) than gained it (7% for both the cancer and stromal cells). Despite this loss, COMP expression in cancer and stromal cells in LNM was associated with progression-free and overall survival from the date of metastasis as well as from the primary breast cancer diagnosis. These findings suggest that COMP may have a role in the intravasation of the tumor cells from the primary tumor already at the time of initial breast cancer diagnosis. These data are in accordance with published studies showing that the expression of COMP by cancer cells induced the epithelial to mesenchymal transition [25]. In addition, COMP expression in primary tumors correlated with liver metastases confirming previous observations [16]. COMP is a crucial cartilage ECM component binding mainly to collagens [26,27]. The liver, lymph nodes, and other collagen-rich tissues could be providing COMP-expressing cancer cells a better locus for metastases. COMP is not only binding to collagen but also other main components of the ECM [3,28], making the hypothesis more complicated. Further studies are needed to thoroughly dissect the relations between ECM composition and COMP expression by the metastatic cancer cells.

High expression of COMP in the stroma of LNM correlated with visceral metastases and the presence of lung metastases. LNM in breast cancer patients are regional metastases that are commonly identified at the time of initial cancer diagnosis. In contrast, DM are found in later, more advanced stages of the disease. One may hypothesize that COMP expression in LNM contributes to the development of distant metastases either by affecting the intravasation of the cancer cells to the bloodstream or the lymphatic system [29] or by providing an advantage to the cancer cells to spread to collagen-rich metastatic locus.

It is clear that breast cancer cells can express COMP since we detected it in an intracellular location when using IHC. Further, COMP is deposited in the stroma of the tumors, but future studies must be designed to address the source of COMP in the stroma; this could be fibroblasts as these were previously shown to express COMP under certain conditions [2]. The initial dogma that the ECM proteins are only secreted by the stromal cells in the tumor microenvironment has been disputed. Recent studies addressing this question found that the source of ECM could be either the stroma cells or the cancer cells [30,31]. Interestingly, in one of these studies COMP has been predicted to be expressed by the cancer cells rather than the stromal cells [30].

One weakness of the current study lies in the limited number of patients that were included, and, therefore, further studies including larger cohorts will be needed to fine-tune conclusions on how to best use COMP as biomarker in the management of breast cancer. The main limitation of the study is the lack of a group of non-metastatic patients. Moreover, a cohort including all patients with metastatic breast cancer is heterogeneous in the sense that the underlying biology is different between the subtypes, and patients vary regarding the extent of the metastatic disease and the therapy that was received. Lastly, for some patients, tissue material was not collected from all of the metastatic sites.

Taken together, COMP emerges as a biomarker of advanced breast cancer progression, especially in ER-positive and HER2-positive patients.

## 5. Conclusions

High COMP expression in lymph node metastases in breast cancer has prognostic implications suggesting that COMP is involved in the progression to a more aggressive metastatic disease. The serum level of COMP appears to be a valuable marker of breast cancer progression as it could distinguish ER or HER2 positive patients with advanced disease. Patients with ER- and HERs-positive MBC and an increased serum level of COMP could potentially benefit from applying more aggressive therapeutic strategies.

## Figures and Tables

**Table 1:** S-COMP levels (high/low) in relation to clinicopathological variables in patients with metastatic breast cancer.

Variables	All Patients, *n* = 141	BL COMP Levels Low (<11.67), *n* = 71	BL COMP Levels High (≥11.67), *n* = 70	*p*-Value
Age at MBC diagnosis				
<65 years	67	40	27	0.04 ^c^
≥65 years	74	31	43	
Metastasis-free interval (years)				
0	29	9	20	0.03 ^c^
>0–3	26	20	6	
>3	86	42	44	
BL ECOG				
0	79	46	33	0.10 ^b^
1	36	13	23	
2	22	10	12	
Unknown	4	2	2	
PT hist subtype				
Ductal	104	57	47	0.01 ^c^
Lobular	26	7	19	
Unknown	11	7	4	
PT NHG				
I	12	9	3	0.41 ^b^
II	59	24	35	
III	42	28	14	
Unknown	28	10	18	
PT T (size)				
T1	50	30	20	0.17 ^b^
T2	47	19	28	
T3	18	13	5	
T4	17	5	12	
Unknown	9	4	5	
PT node status				
Neg	41	26	15	0.13 ^c^
Pos	80	39	41	
Unknown	20	6	14	
Breast cancer subtype ^a^				
ER+HER2−	98	46	52	0.04 ^b^
HER2+	15	4	11	
ER−HER2−	25	19	6	
Unknown	3	1	2	
No of metastatic sites				
<3	99	49	50	0.75 ^c^
≥3	42	22	20	
Bone metastasis				
Yes	98	42	56	0.007 ^c^
No	43	29	14	
Liver metastasis				
Yes	39	19	20	0.81 ^c^
No	102	52	50	
Lung metastasis				
Yes	46	31	15	0.005 ^c^
No	95	40	55	
Visceral metastasis ^d^				
Yes	83	43	40	0.68 ^c^
No	58	28	30	
Treatment				
Chemotherapy	64	30	34	0.03 ^c^
Endocrine therapy	58	38	20	
HER2-directed therapy	13	4	9	
CTC count				
<5	67	44	23	<0.001 ^c^
≥5	72	26	46	
unknown	2			
CTC-cluster				
Yes	13	1	12	0.001 ^c^
No	126	69	57	
Unknown	2	1	1	
COMP score cancer cells (IHC)				
0	17	9	8	0.13 ^b^
1	14	7	7	
2	9	1	8	
3	11	3	8	
Unknown	90	51	39	
COMP score stroma (IHC)				
0	4	4	0	0.01 ^b^
1	17	9	8	
2	17	3	14	
3	13	4	9	
Unknown	90	51	39	

Abbreviations: BL baseline, MBC metastatic breast cancer, ECOG Eastern Cooperative Oncology Group, PT primary tumor, NHG Nottingham histological grade, ER estrogen receptor, HER2 human epidermal growth receptor 2, CTC circulating tumor cells, IHC immunohistochemistry. ^a^ Breast cancer subtype was primarily derived from immunohistochemical staining of the metastasis. If no information was available from the metastasis, the subtype was derived by staining of the primary tumor. ^b^ *p* value from Pearson’s chi-squared test for trend. ^c^ *p* value from Pearson’s chi-squared test. ^d^ Visceral metastasis was defined as presence of lung, liver, brain, peritoneal, and/or pleural involvement.

**Table 2:** Shifts in COMP expression from primary tumors to lymph node metastases and distant metastases, evaluated with McNemar’s analyses.

Location	COMP Expression in Cancer Cells N (%)	*p*	COMP Expression in Stroma N (%)	*p*
PT+/LNM+	27 (47)	0.08	33 (58)	0.049
PT+/LNM−	12 (21)		13 (23)	
PT−/LNM+	4 (7)		4 (7)	
PT−/LNM−	14 (25)		7 (12)	
Total	57 (100)		57 (100)	
PT+/DM+	23 (55)	0.77	32 (76)	0.51
PT+/DM−	7 (17)		3 (7)	
PT−/DM+	5 (12)		6 (14)	
PT−/DM−	7 (17)		1 (2)	
Total	42 (100)		42 (100)	
LNM+/DM+	6 (23)	0.80	18 (69)	0.13
LNM+/DM−	7 (27%)		1 (4)	
LNM−/DM+	9 (35)		6 (23)	
LNM−/DM−	4 (15)		1 (4)	
Total	26		26	

Abbreviations: PT primary tumor, LNM lymph node metastasis, DM distant metastasis.

**Table 3:** COMP expression in primary tumors and lymph node metastases in relation to clinicopathological variables in patients with metastatic breast cancer. Bold indicates *p* values < 0.05.

	COMP PT Cancer Cells					COMP PT Stroma					COMP LNM Cancer Cells					COMP LNM Stroma				
Variables	0	1	2	3	*p*	0	1	2	3	*p*	0	1	2	3	*p*	0	1	2	3	*p*
Age <65 >65	24	14	14	6	**0.005**	11	15	22	10	0.15	13	6	4	5	0.39	12	3	11	2	0.13
	13	11	21	14		7	14	22	16		16	8	6	11		10	9	13	9	
ECOG 0 1 2	23	15	22	7	0.10	11	17	26	13	0.41	20	10	4	10	0.09	17	7	14	6	**0.02**
	8	7	9	7		4	9	11	7		6	3	4	3		4	4	5	3	
	5	3	4	6		3	3	6	6		1	1	2	3		0	0	5	2	
MFI 0years 0–3years >3years	4	3	9	7	0.22	0	2	12	9	**0.007**	1	2	1	2	0.75	1	0	3	2	0.31
	12	5	4	2		5	7	8	3		6	4	3	1		5	3	4	2	
	21	17	22	11		13	20	24	14		22	8	6	13		16	9	17	7	
Subtype ductal	25	21	23	16	0.78	12	20	31	22	0.50	17	11	5	13	0.51	12	7	19	8	0.28
lobular	7	3	7	3		4	6	8	2		10	2	3	2		8	5	3	1	
other	4	1	5	1		1	3	5	2		2	0	2	1		2	0	1	2	
PT T1	13	8	13	4	0.84	6	10	14	8	0.90	9	2	2	3	0.81	6	3	3	4	0.37
T2	7	10	13	11		5	10	15	11		9	6	6	8		4	8	12	5	
T3	10	3	5	0		6	3	8	1		7	2	1	3		7	1	5	0	
T4	5	3	2	5		1	4	5	5		3	2	1	2		4	0	2	2	
Node neg Node pos	10	8	9	4	0.87	5	9	10	7	0.84	x	x	x	X	X	x	x	x	X	x
	24	16	21	11		13	19	26	14		x	x	x	x		x	x	x	x	
NHG I II III	5	0	3	0	0.44	2	1	3	2	0.68	4	0	1	0	0.12	2	2	1	0	0.31
	14	11	15	7		5	15	19	8		17	5	6	10		11	8	13	6	
	13	8	12	6		8	10	13	8		4	7	3	5		6	2	7	4	
PT ER- ER+	7	6	4	2	0.25	3	6	6	4	0.67	3	2	0	2	0.84	2	1	2	2	0.56
	30	17	31	17		15	21	38	21		24	10	10	16		19	11	19	9	
PT HER2- HER2+	30	16	26	14		15	22	31	18		18	9	6	12		14	10	14	7	
	1	3	4	3		0	1	6	4		1	3	3	1		2	0	5	1	
Mets <3 >=3	24	21	22	10	0.21	10	22	32	13	0.51	21	9	8	9	0.41	16	10	12	9	0.58
	13	4	13	10		8	7	12	13		8	5	2	7		6	2	12	2	
CTC <5 >=5	15	14	15	10	0.68	8	11	20	15	0.26	13	7	3	5	0.28	9	4	11	4	0.91
	22	11	20	10		10	18	24	11		16	6	7	11		13	8	12	7	
Cluster neg	29	21	28	18	0.40	14	24	36	22	0.63	21	12	6	11	0.56	15	10	17	8	0.79
pos	8	4	7	2		4	5	8	4		8	1	4	5		7	2	6	3	
Visc no	19	11	13	6	0.09	9	15	18	7	0.07	14	7	1	6	0.21	12	8	4	4	**0.03**
yes	18	14	22	14		9	14	26	19		15	7	9	10		10	4	20	7	
Bone-only no	27	19	29	15	0.58	15	18	35	22	0.38	23	10	9	12	0.97	18	7	21	8	0.95
yes	10	6	6	5		3	11	9	4		6	4	1	4		4	5	3	3	
Lung met no	26	19	23	12	0.37	11	21	33	15	0.79	23	10	7	10	0.23	19	11	12	8	**0.04**
yes	11	6	12	8		7	8	11	11		6	4	3	6		3	1	12	3	
Liver mets no	31	19	23	11	**0.01**	15	26	27	16	**0.01**	23	12	4	10	0.07	17	10	17	5	0.09
yes	6	6	12	9		3	3	17	10		6	2	6	6		5	2	7	6	
Bone mets no	16	8	11	5	0.16	9	6	17	8	0.60	9	5	6	3	0.78	8	3	7	5	0.82
yes	21	17	24	15		9	23	27	18		20	9	4	13		14	9	17	6	

Abbreviations: PT primary tumor, LNM lymph node metastasis, ECOG eastern cooperative oncology group, MFI metastasis-free interval, NHG Nottingham histological grade, ER estrogen receptor, HER2 human epidermal growth factor receptor 2, CTC circulating tumor cells. Visceral metastasis was defined as the presence of lung, liver, brain, peritoneal, and/or pleural involvement. *p*-values from Pearson’s chi-squared test for trend.

## Data Availability

The data presented in this study are available on request from the corresponding author. The data are not publicly available due to privacy and ethical reasons.

## Supplementary Materials

The following are available online at https://www.mdpi.com/article/10.3390/cancers13235876/s1, Table S1: COMP expression in distant metastases.
