# Supplementary material for: High Levels of Expression of Cartilage Oligomeric Matrix Protein in Lymph Node Metastases in Breast Cancer Are Associated with Reduced Survival
Source: Cancers (Basel). 2021 Nov 23;13(23):5876. doi: 10.3390/cancers13235876 (PMC8656813; doi:10.3390/cancers13235876)
Supplement: Supplementary file 1 [file cancers-13-05876-s001.zip › cancers-1482542-supplementary.pdf]

Supplementary materials: High Levels of Expression of Cartilage Oligomeric Matrix Protein in Lymph Node Metastases in Breast Cancer Are Associated with Reduced Survival

Konstantinos S. Papadakos, Catharina Hagerling, Lisa Rydén, Anna-Maria Larsson and Anna M. Blom

Table S1. COMP expression in distant metastases in relation to clinicopathlogical variables in patients with metastatic breast cancer.

	COMP DM Cancer Cells					COMP DM Stroma				
Variables	0	1	2	3	*P*	0	1	2	3	*P*
Age <65	11	7	2	6	0.33	2	9	7	8	0.76
>65	6	8	7	5		2	8	11	5	
MFI 0y	1	4	2	3	0.17	0	4	3	3	0.45
0-3 y	3	0	1	2		1	1	1	3	
>3y	13	11	6	6		3	12	14	7	
Subtype ductal	9	10	8	5	0.88	2	11	13	6	0.54
Lobular	7	4	1	6		2	5	4	7	
PT T1	6	6	5	3	0.99	2	4	10	4	0.89
T2	6	5	1	6		0	9	3	6	
T3	4	1	0	1		2	1	2	1	
T4	1	1	2	1		0	1	3	1	
Node pos	13	10	3	9	0.74	3	12	12	8	0.75
Node neg	3	3	3	2		1	3	4	3	
NHG I	1	3	0	1	0.65	0	2	2	1	0.90
II	13	3	4	6		4	8	7	7	
III	2	4	2	2		0	4	4	2	
PT ER-	2	0	2	2	0.36	0	1	3	2	0.26
ER+	15	11	7	8		4	14	13	10	
PT HER2-										
HER2+										
Mets <3	12	10	7	11	0.07	3	11	14	12	0.13
>=3	5	5	2	0		1	6	4	1	
CTC <5	4	9	4	4	0.51	1	9	6	5	0.84
>=5	13	6	4	7		3	8	12	7	
Cluster neg	9	13	6	9	0.13	2	13	13	9	0.62
pos	8	2	2	2		2	4	5	3	
Visc no	10	7	4	8	0.59	3	7	11	8	0.61
yes	7	8	5	3		1	10	7	5	
Bone-only no	10	11	6	5	0.51	3	12	10	7	0.26
yes	7	4	3	6		1	5	8	6	
Lung met no	12	14	8	10	0.16	3	14	15	12	0.37
yes	5	1	1	1		1	3	3	1	
Liver mets no	13	10	7	9	0.67	4	11	14	10	1.0
yes	4	5	2	2		0	6	4	3	
Bone mets no	3	2	1	2	0.96	1	3	2	2	0.63
yes	14	13	8	9		3	14	16	11	
